# Structural investigations into a new polymorph of F_4_TCNQ: towards enhanced semiconductor properties

**DOI:** 10.1107/S2053229621006252

**Published:** 2021-06-28

**Authors:** Natalie T. Johnson, Michael R. Probert, Paul G. Waddell

**Affiliations:** a Chemistry, School of Natural and Environmental Sciences, Newcastle University, Bedson Building, Edward’s Walk, Newcastle Upon Tyne NE1 7RU, UK

**Keywords:** polymorphism, F_4_TCNQ, charge transfer, pairwise inter­action energies, crystal structure, semiconductor, quinodimethane

## Abstract

During the course of research into the structure of 2,3,5,6-tetra­fluoro-7,7,8,8-tetra­cyano­quinodi­methane (F_4_TCNQ), C_12_F_4_N_4_, an important com­pound in charge-transfer and organic semiconductor research, a previously unreported polymorph of F_4_TCNQ was grown concomitantly with the known polymorph from a saturated solution of di­chloro­methane. The structure was elucidated using single-crystal X-ray diffraction and it was found that the new polymorph packs with mol­ecules in parallel layers, in a similar manner to the layered structure of F_2_TCNQ. The structure was analysed using Hirshfeld surface analysis, fingerprint plots and pairwise inter­action energies, and com­pared to existing data. The structure of a toluene solvate of F_4_TCNQ is also reported.

## Abstract 2

A new polymorph of 2,3,5,6-tetra­fluoro-7,7,8,8-tetra­cyano­quinodi­methane (F_4_TCNQ) has been characterized using single-crystal X-ray diffraction and is presented in com­parison with the previously reported structure of the com­pound.

## Introduction   

**Figure 1:**
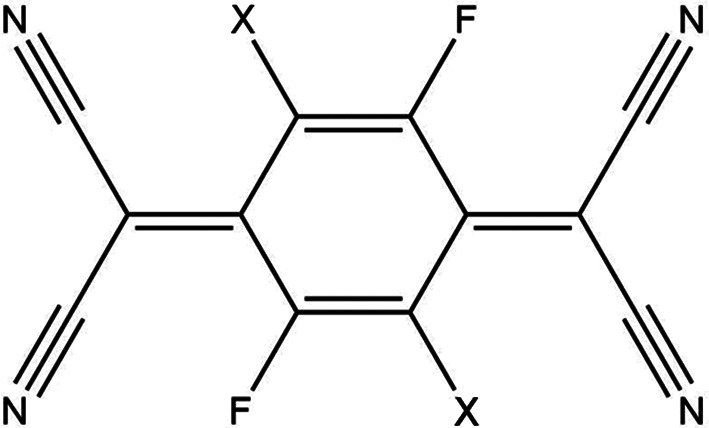
The structure of F_*n*_TCNQ, where *X* = H for F_2_TCNQ and *X* = F for 2,3,5,6-tetra­fluoro-7,7,8,8-tetra­cyano­quinodi­methane (F_4_TCNQ)

2,3,5,6-Tetra­fluoro-7,7,8,8-tetra­cyano­quinodi­methane (F_4_TCNQ; Fig. 1 ▸) was first characterized using X-ray crystallography by Emge *et al.* (1981 ▸) [Cambridge Structural Database (CSD; Version 5.41 of November 2019; Groom *et al.*, 2016 ▸) refcode BAKPAE] and has been reported as both a homomolecular structure (BAKPAE01–03; Krupskaya *et al.*, 2015 ▸; Salzillo *et al.*, 2016 ▸; Shukla *et al.*, 2019 ▸) and the coformer in various cocrystals, with 229 instances of such cocrystals in the CSD. F_4_TCNQ is of particular inter­est to materials scientists given its high electron affinity (Gao & Kahn, 2001 ▸) and stable anionic form, which make it suitable for use as a p-type dopant for a range of semiconductors (Gao & Kahn, 2001 ▸; Pingel *et al.*, 2012 ▸; Cochran *et al.*, 2014 ▸). These properties have also given rise to the use of F_4_TCNQ as an electron acceptor in charge-transfer com­plexes (Sutton *et al.*, 2016 ▸; Hu *et al.*, 2017 ▸; Fujii & Yamakado, 2018 ▸).

The family of F_*n*_TCNQ com­pounds (*n* = 0, 2, 4) was identified as an important series of mol­ecules for the understanding of electron transport in crystals, due to the dif­ferences in electronic properties across the series of similar mol­ecules (Krupskaya *et al.*, 2015 ▸). While F_4_TCNQ (*n* = 4) and TCNQ (*n* = 0) were found to have low electron mobility (0.1 and 0.2 cm^2^ V^−1^ s^−1^ at room temperature, respectively), F_2_TCNQ (2,5-di­fluoro-7,7,8,8-tetra­cyano­quinodi­methane, C_12_H_2_F_2_N_4_; Fig. 1 ▸) was found to have a much higher electron mobility of 6–7 cm^2^ V^−1^ s^−1^ at room temperature (and up to 25 cm^2^ V^−1^ s^−1^ at 150 K). Band-like electron transport, where the electron mobility increases upon lowering the temperature, has been observed in F_2_TCNQ but not in the other com­pounds.

**Figure 2:**
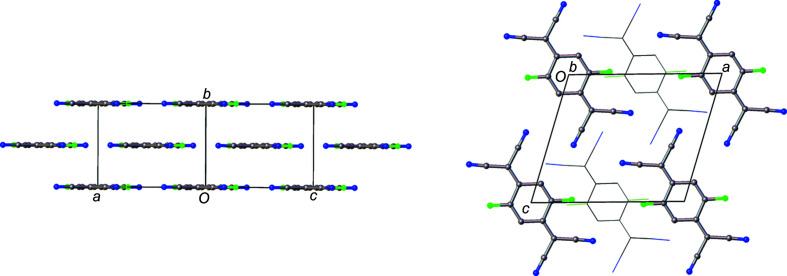
The structure of F_2_TCNQ (CSD refcode BERZON03; Krupskaya *et al.*, 2015 ▸) highlighting the relationship between mol­ecules in adjacent layers.

**Figure 3:**
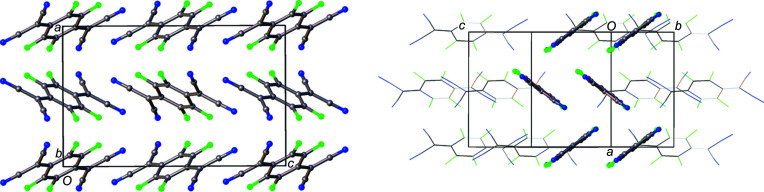
The structure of F_4_TCNQ polymorph I, highlighting the relationship between mol­ecules in adjacent layers (left) and the herringbone arrangement of mol­ecules along [100] (right).

Krupskaya *et al.* (2015 ▸) postulated that the difference in the crystal structures of the com­pounds could be the cause of the difference in electron mobility across the F_*n*_TCNQ family. Solid-state structure is extremely important for electron mobility (Wang *et al.*, 2012 ▸; Coropceanu *et al.*, 2007 ▸) and F_2_TCNQ has a markedly different structure to the other members of the family. In F_2_TCNQ (BERZON03; Krupskaya *et al.*, 2015 ▸), the mol­ecules pack in a layered structure with mol­ecules in adjacent (010) layers coplanar with each other (Fig. 2 ▸). This is different to that of the reported structure of F_4_TCNQ (BAKPAE03; Shukla *et al.*, 2019 ▸), where the mol­ecules are packed in a herringbone manner (Fig. 3 ▸).

Further study of this family of com­pounds also attributed the high electron mobility of F_2_TCNQ to its crystal structure (Chernyshov *et al.*, 2017 ▸; Sosorev, 2017 ▸; Ji *et al.*, 2018 ▸; Sosorev *et al.*, 2018 ▸). According to these studies, electron motility in the solid state is affected by the number of mol­ecules in the reduced unit cell of the crystal structure, with lower values prohibiting inter­molecular vibrations according to the rigid mol­ecule approximation (Sosorev *et al.*, 2019 ▸). The absence of these modes results in a weakening of the electron–phonon inter­action; a smaller electron–phonon inter­action can indicate a lesser degree of charge localization in the structure, and hence greater electron mobility (Chernyshov *et al.*, 2017 ▸). As F_2_TCNQ crystallizes with one mol­ecule in its reduced unit cell (com­pared to two and four mol­ecules in those of TCNQ and F_4_TCNQ), it can be expected to exhibit greater electron motility as a result.

Raman spectroscopy has been used to investigate electron–phonon inter­actions in the crystal structure, where charge mobility has been shown to be related to the value of the lowest vibrational frequency mode (Fratini *et al.*, 2016 ▸). The lowest vibrational mode for F_2_TCNQ was found to be almost double the values for TCNQ and F_4_TCNQ (polymorph I) (Chernyshov *et al.*, 2017 ▸; Sosorev *et al.*, 2018 ▸). Theoretical calculations have shown F_2_TCNQ to have a three-dimensional charge carrier network (Ji *et al.*, 2018 ▸; Sosorev, 2017 ▸), which is attributed to its high charge mobility and band-like electron transport, while for F_4_TCNQ and TCNQ, the charge mobility is hindered by the mol­ecular structure and strong thermal disorder.

In the process of growing high-quality single crystals of F_4_TCNQ, an additional polymorph of F_4_TCNQ (polymorph II) was found that exhibits a layered structure similar to the structure of F_2_TCNQ. The structure of this new polymorph was measured using single-crystal X-ray diffraction and com­pared to the known structures of F_4_TCNQ using Hirshfeld surface analysis, fingerprint plots and pairwise inter­action energies. The structure is also com­pared to the previously published structure of F_2_TCNQ (BERZON03; Krupskaya *et al.*, 2015 ▸), as the data were measured at 100 K, the same temperature as the F_4_TCNQ studies reported herein. When crystallized from toluene, a toluene–F_4_TCNQ solvate was obtained, the structure of which is also presented.

## Experimental   

### Crystallization   

F_4_TCNQ was purchased from Apollo Scientific as a solid with 97% purity and was used without further purification. Crystals suitable for single-crystal X-ray diffraction were grown *via* slow evaporation of the solvent from solutions of the com­pound in aceto­nitrile, di­chloro­methane (DCM) and toluene. All crystal formation took place within 24–48 h.

**Figure 4:**
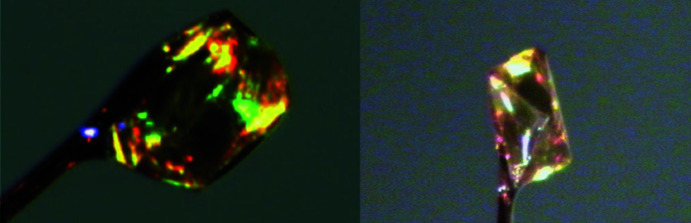
A crystal of F_4_TCNQ polymorph I as mounted on the diffractometer (left). The octa­hedral crystal of F_4_TCNQ polymorph II (right).

The crystals of F_4_TCNQ grown from saturated solutions of both aceto­nitrile and DCM were found to be homomolecular. Crystallization from aceto­nitrile yielded only crystals of the previously reported structure (polymorph I), which form as yellow crystals with a regular block-like morphology, whereas in DCM, single crystals exhibiting two different morphologies were observed to form concomitantly, *i.e.* cubic crystals of poly­morph I alongside octa­hedral crystals (Fig. 4 ▸). The octa­hedral crystals are the same yellow colour as those of polymorph I but yield a drastically different crystal structure (polymorph II).

F_4_TCNQ crystallizes from toluene as a toluene–F_4_TCNQ solvate in the form of red needles.

### Data collection   

Crystals of polymorphs I and II and the toluene solvate were analysed using single-crystal X-ray diffraction. The crystal of polymorph I selected was grown from a saturated solution of aceto­nitrile, which produced larger and more abundant crystals of this polymorph than were observed in similar DCM solutions. Although the structure of polymorph I has been elucidated previously at 100 K (Shukla *et al.*, 2019 ▸), the structure was redetermined in a manner more consistent with the data collection for polymorph II to allow for a more direct com­parison between the two structures.

Crystals of polymorphs I and II were cooled slowly to 100 K at a rate of 1 K min^−1^ using an Oxford Cryosystems N_2_ cryostream cooler on a Bruker D8 Venture diffractometer. X-rays were generated using an Incoatec IµS 3.0 Ag source (Ag *K*α, λ = 0.56086 Å). The data collected were prone to white radiation contamination (as described in Storm *et al.*, 2004 ▸); therefore, a 150 µm aluminium filter was included to remove this white radiation before the beam impinged on the sample (Macchi *et al.*, 2011 ▸). The diffraction pattern was measured on a Photon II CPAD detector using the shutterless operation mode with a sample-to-detector distance of 65 mm.

A crystal of F_4_TCNQ–toluene was measured using Cu radiation (Cu *K*α, λ = 1.54184 Å) at 150 K on a Rigaku Oxford Diffraction Xcalibur Atlas Gemini diffractometer equipped with an Oxford Cryosystems N_2_ open-flow cooling device.

### Refinement   

Crystal data, data collection and structure refinement details are summarized in Table 1 ▸. H atoms were placed with idealized geometry, with *U*_iso_(H) values constrained to be an appropriate multiple of the *U*_eq_ value of the parent atom. In the toluene solvent structure of F4TCNQ, the toluene molecule has been modelled as disordered over two sites across a centre of symmetry. The occupancies of the two parts were constrained to be 0.5 and the atomic displacement parameters were restrained. The geometry of the toluene molecule was also restrained.

## Results and discussion   

### Comparison of F_4_TCNQ polymorphs   

Table 1 ▸ shows a summary of the experimental details for the crystallographic data from polymorphs I and II and the toluene solvate. Both forms of F_4_TCNQ are very stable under ambient conditions; over a period of six months, no inter­conversion was observed between forms. Both polymorphs crystallize in the ortho­rhom­bic crystal system and centrosymmetric space groups. The atoms in F_4_TCNQ in polymorph II sit on special positions in the unit cell, effectively a horizontal mirror plane, thereby halving the number of atoms in the asymmetric unit relative to polymorph I.

The mol­ecular geometry of F_4_TCNQ is almost identical in polymorphs I and II (with no statistically different bond lengths or angles). This is unsurprising owing to the planarity and conformational inflexibility of F_4_TCNQ that is due to the high degree of conjugation within the mol­ecule. However, despite their similar mol­ecular structures, the packing of the mol­ecules in the two crystal structures is markedly different.

**Figure 5:**
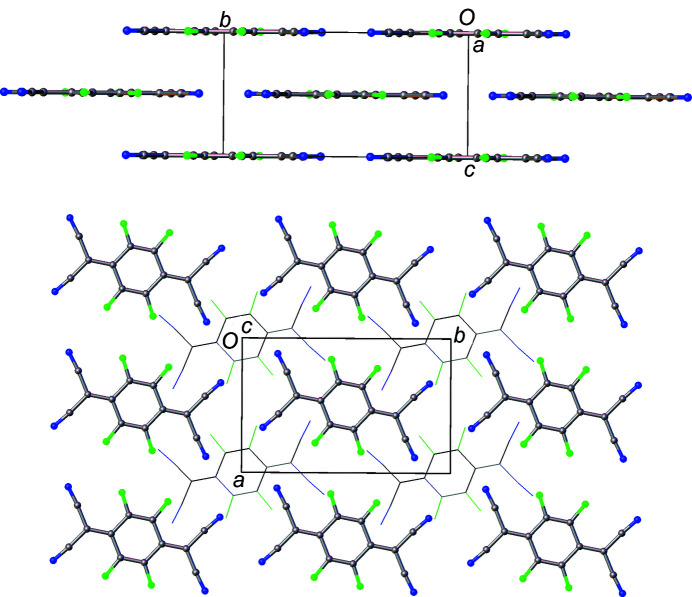
View down the [100] (top) and [001] (bottom) crystallographic planes of F_4_TCNQ polymorph II.

The F_4_TNCQ mol­ecules in polymorph II are arranged to form layers coplanar with the crystallographic [001] plane and, as a result, the mol­ecules in each layer are arranged coplanar to those in adjacent layers (Fig. 5 ▸) at a coplanar distance of *ca* 2.98 Å. Within a layer, the mol­ecules are related by crystallographic translations in the [100] and [010] directions. The orientation of the mol­ecules in adjacent layers alternates with respect to the previous layer in a manner consistent with the symmetry of the *n*-glides in the [101] and [011] directions.

**Figure 6:**
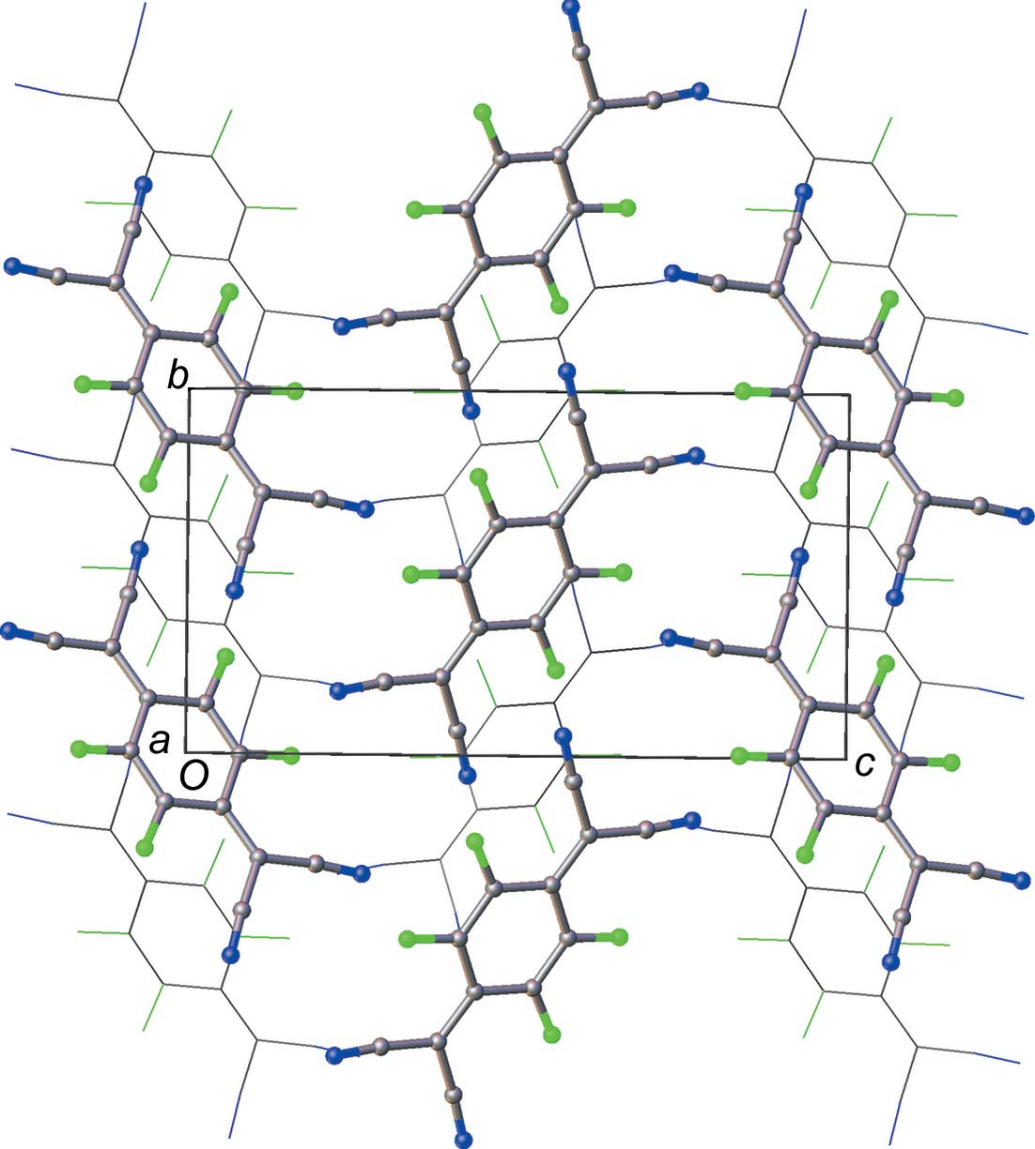
A view of polymorph I with adjacent layers visible in the [100] direction.

Layers of mol­ecules can also be seen in polymorph I, but the mol­ecules are not arranged coplanar to each other. In this case, the mol­ecules within the structure can be described as packing in a herringbone pattern, as illustrated in Fig. 3 ▸, an alternative view of the crystal structure along the [011] plane. Adjacent mol­ecules, drawn using a wireframe model, are also arranged in a herringbone formation, but at 90° to the herringbone chain highlighted in the figure. Along the [100] direction in Fig. 6 ▸, mol­ecules are arranged in alternating orientations, which also form a herringbone motif.

### Hirshfeld surfaces   

Hirshfeld surfaces were calculated for the two polymorphs (Spackman & Jayatilaka, 2009 ▸; McKinnon *et al.*, 2007 ▸). The normalized distance (*d*_norm_) between the closest external and inter­nal atoms to any point on the surface is represented by the colour on the surface. A pair of atoms with *d*_norm_ less than the van der Waals radius of the atoms is shown in red and could indicate a close contact between those two atoms. These close contacts are important as they could indicate favourable inter­actions within the crystal, which could direct the packing of the mol­ecules in the structure or influence the properties of the crystal (Bernstein, 1993 ▸).

**Figure 7:**
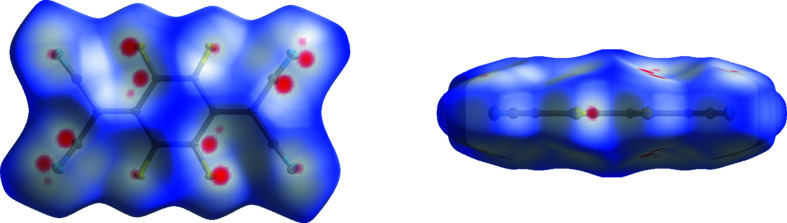
Hirshfeld surface calculated for a mol­ecule of F_4_TCNQ in polymorph II.

**Figure 8:**
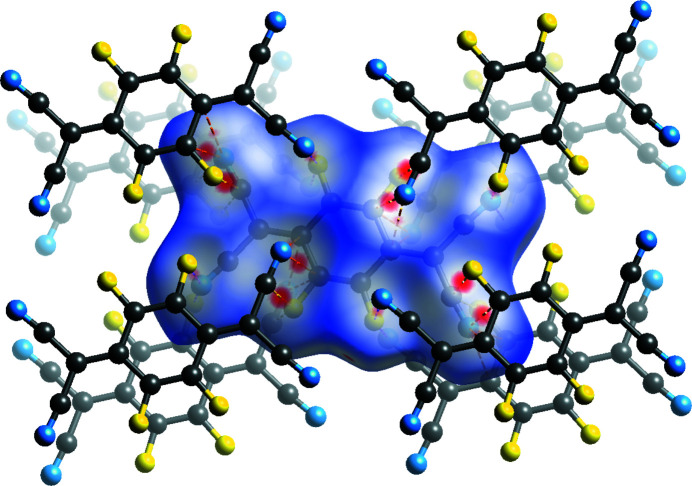
Hirshfeld surface of polymorph II, showing close contacts between atoms in adjacent layers, with adjacent mol­ecules shown.

The surface for polymorph II (Fig. 7 ▸) indicates that the majority of close contacts occur between mol­ecules in adjacent layers. These occur in two different motifs: motif 1 between pairs of C⋯F and C⋯N inter­actions corresponding to close contacts between the atoms of the C—F bond of one mol­ecule and those of the C≡N group of an adjacent mol­ecule in another layer (the atoms involved in this motif produce the most prominent red spots on the Hirshfeld surface), with a distance of 3.1197 (2) Å between the centroids of these two bonds; and motif 2 between only the terminal atoms of the aforementioned bonds, with the N and F atoms (Fig. 8 ▸) at a distance of 2.9885 (2) Å. Half of the C≡N and C—F atoms in a mol­ecule exhibit close contacts of motif 1 only and the other half exhibit motif 2 only, with the same pattern of close contacts observed to form to both adjacent layers.

The arrangement of the atoms of motif 1 form a four-membered ring of close contacts. There are only four other non-organometallic structures in the CSD that contain this motif of close contacts (Wiscons *et al.*, 2018 ▸; Fan & Yan, 2014 ▸; Ishida *et al.*, 2014 ▸; Sutton *et al.*, 2016 ▸). Two of these also contain F_4_TCNQ, and the motif occurs only between F_4_TCNQ mol­ecules in the structure (Wiscons *et al.*, 2018 ▸; Sutton *et al.*, 2016 ▸).

**Figure 9:**
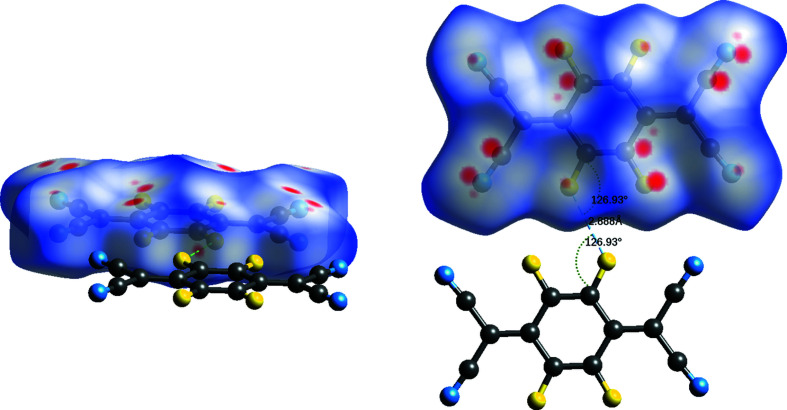
Hirshfeld surface of polymorph II, showing close contacts between atoms in the same layer, with adjacent mol­ecules shown.

There is only one type of close contact between mol­ecules in the same layer, which forms between two F atoms in adjacent mol­ecules (Fig. 9 ▸), with a distance of 2.8881 (7) Å, which is within the sum of the van der Waals radii (Alvarez, 2013 ▸). This is observed for two of the four F atoms in the mol­ecule. Halogen bonding rules would suggest that this is a type-II contact, occurring because of the proximity of the F atoms in the structure, rather than due to the formation of a stabilizing/favourable inter­action (Metrangolo & Resnati, 2013 ▸).

**Figure 10:**
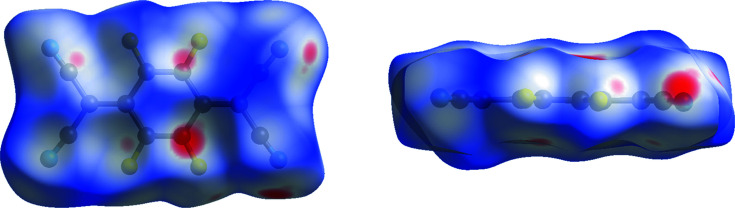
The Hirshfeld surface of polymorph I.

In contrast, there are fewer close contacts between mol­ecules in polymorph I [22 *versus* 34 from one mol­ecule, when totalled from those identified by *Mercury* (Macrae *et al.*, 2020 ▸)]. Most of the close contacts observed in polymorph II are not present in this arrangement – except for the F⋯N (motif 2) close contact (Fig. 10 ▸, and Fig. S1 in the supporting information shows the close contacts with adjacent mol­ecules).

### Fingerprint plots   

**Figure 11:**
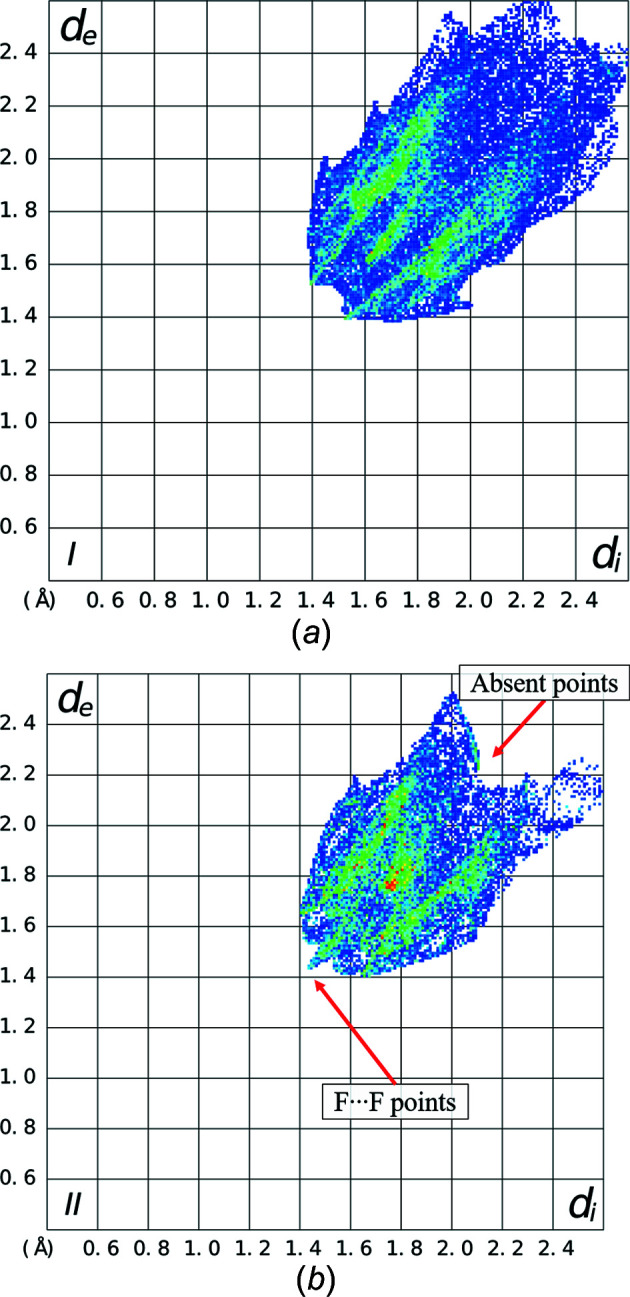
Fingerprint plots for polymorphs I and II.

Fingerprint plots (Spackman & McKinnon, 2002 ▸) result from the calculation of the distance to the closest inter­nal and external atom for each point on the Hirshfeld surface, with the values displayed graphically. They have been used to com­pare polymorph structures by highlighting differences in the closest atomic contacts in the structures (McKinnon *et al.*, 2007 ▸). Those created for polymorphs I and II (Fig. 11 ▸) further illustrate the differences in packing between the two forms. In polymorph II, there are some additional points along the diagonal of the graph at short distances, which are a result of like–like F⋯F contacts, contacts between equivalent F atoms externally and inter­nally of the Hirshfeld surface. In polymorph I, F atoms in adjacent mol­ecules do not approach as closely as observed in polymorph II. This is evident in Fig. S2 (see supporting information), a version of the fingerprint plots where only points relating to F⋯F contacts are displayed in colour.

### Energy com­parisons   

To further com­pare polymorphs, pairwise inter­action energies were calculated using *CrystalExplorer* (Turner *et al.*, 2014 ▸, 2017 ▸). As both polymorphs form concomitantly in DCM, but only polymorph I has been reported in the literature, there may be an energetic preference for one polymorph over another. Pairwise inter­action energies were calculated for a central mol­ecule to surrounding mol­ecules within a radius of 3.8 Å and consist of scaled values for electrostatic, repulsive, polarization and dispersion contributions to the total inter­action energy (*E*_tot_) using the [B3LYP/6-31G(d,p)] energy model. The tables of values are included in the supporting information (Tables S2 and S3). These values can be used to com­pute an approximate average energy of the structure, and thus indicate if one polymorph is more stable than another. Energy frameworks for F_4_TCNQ polymorph I and F_2_TCNQ have been discussed previously by Shukla *et al.* (2019 ▸).

**Figure 12:**
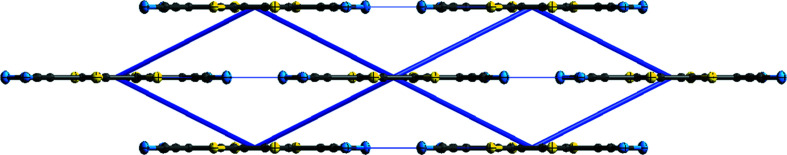
Energy framework for polymorph II of F_4_TCNQ calculated using *CrystalExplorer17.5*. The lines between mol­ecules indicate the relative size of the pairwise energies between mol­ecules. *E*_tot_ between mol­ecules in adjacent layers are calculated as −33.3 kJ mol^−1^.

**Figure 13:**
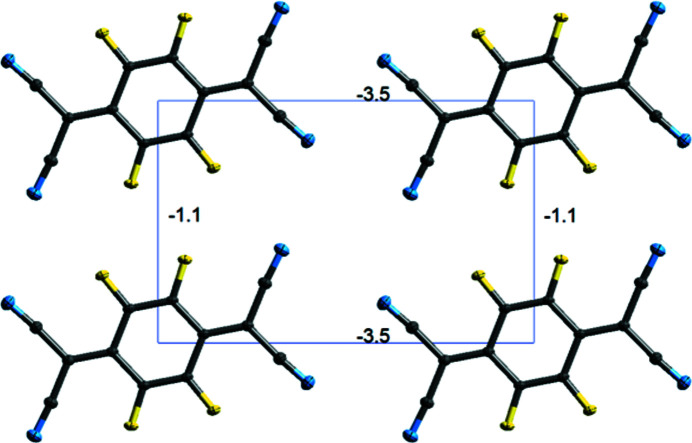
Energy framework for mol­ecules within a layer of polymorph II of F_4_TCNQ, calculated using *CrystalExplorer17.5*.

For polymorph II, there are three different mol­ecule pairs – one from the central mol­ecule to mol­ecules in adjacent layers, and two from mol­ecules within the layers. The energy frame­works created from the calculations show that the largest *E*_tot_, −33.3 kJ mol^−1^, is calculated between mol­ecules in adjacent layers (Fig. 12 ▸). This value is much larger than the contributions between atoms in the same layers, which are less than −5 kJ mol^−1^. Within the layers, there are two different pairs of inter­actions (Fig. 13 ▸) – those that form close F⋯F contacts between mol­ecules and those that do not. Both pairs having positive electrostatic energies, indicating destabilizing contributions from electrostatic inter­actions. It is inter­esting to note that the mol­ecules within the layers that have close F⋯F contacts are calculated as having an overall stabilizing inter­action, albeit small (−1.1 kJ mol^−l^), despite the positive electrostatic energy.

**Figure 14:**
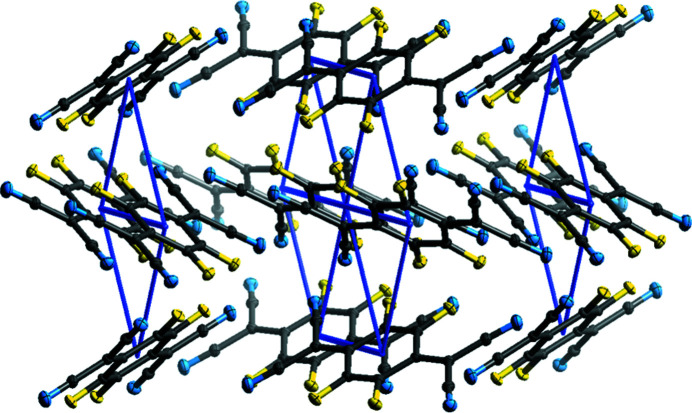
Energy framework of F_4_TCNQ polymorph I, calculated using *CrystalExplorer17.5* (with pairwise energies < 15 kJ mol^−1^ removed for clarity).

In polymorph I, the inter­action energies have less variation. Fig. 14 ▸ shows a view of the energy framework for *E*_tot_. The largest negative values of *E*_tot_ are found for mol­ecules in the same herringbone chain, with the greatest overall being for mol­ecules that are also in the same layer (−34.0 kJ mol^−1^). This value is the largest calculated *E*_tot_ of the two polymorphs. Smaller *E*_tot_ values are calculated between the other sur­rounding mol­ecules. All pairs of mol­ecules have negative calculated electrostatic energies, *E*_ele_.

If mean pairwise energies are calculated by averaging the contributions of the surrounding mol­ecules, we obtain values of −17.85 and −23.03 kJ mol^−1^ for polymorphs I and II, res­pectively (Equation S1 in the supporting information). These values are similar in energy, which is expected in con­comitant polymorphism. It is inter­esting to note that the unreported polymorph II is lower in energy and likely the thermodynamic polymorph, which raises the question of why it has not been reported previously.

Crystallization conditions have been shown to play a role in polymorph formation (Bernstein & Bernstein, 2002 ▸; Isakov *et al.*, 2013 ▸; Tran *et al.*, 2012 ▸). In the previous reported structures of F_4_TCNQ polymorph I that were deposited in the CSD, crystals were grown using vapour transport (Krupskaya *et al.*, 2015 ▸), solution growth (Salzillo *et al.*, 2016 ▸), sublimation (Shukla *et al.*, 2019 ▸) and from a solution of aceto­nitrile (Emge *et al.*, 1981 ▸). The growth of only polymorph I from recrystallizations with aceto­nitrile could suggest an inter­action between the solvent and the mol­ecule which prohibits or makes it less favourable to form the polymorph II. This may be the result of an inter­action between the cyano group of aceto­nitrile with the C—F bond of F_4_TCNQ. A similar ring formed of these inter­actions is seen between aceto­nitrile and a C—F moiety in hexa­kis­(penta­fluoro­phen­yl)[28]hexa­phyrin (LIVHUV; Ishida *et al.*, 2014 ▸). If aceto­nitrile blocks other mol­ecules of F_4_TCNQ from associating with the C—F bond to form the stabilizing four-membered ring close contact motif by inter­acting in that position itself, then other inter­actions may take precedent during crystallization to direct the formation of the structure. If this is indeed the case, then polymorph II is able to form in DCM as the cyano group is absent from the solvent.

### Comparison of polymorph II to F_2_TCNQ   

The reported structure of F_2_TCNQ (Krupskaya *et al.*, 2015 ▸) was analysed in a similar way to the polymorphs of F_4_TCNQ.

**Figure 15:**
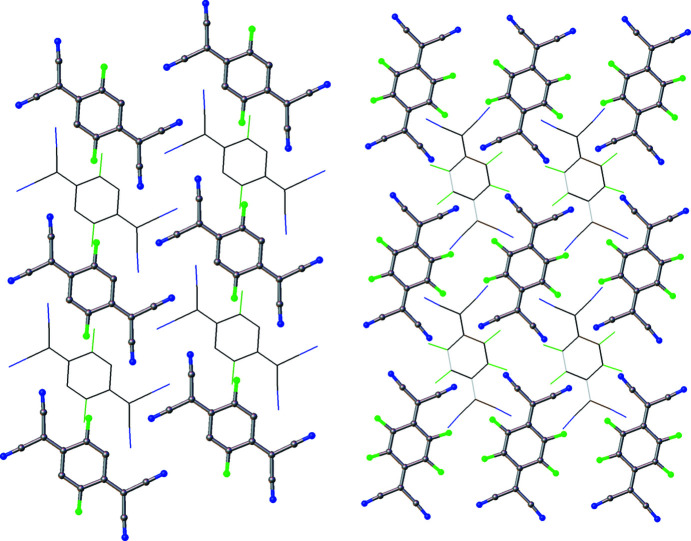
Views of F_2_TCNQ along the [010] axis (left) and of F_4_TCNQ along the [010] axis (right). In F_2_TCNQ, mol­ecules in adjacent layers (drawn with wireframe model) are in the same direction, which is not the case in F_4_TCNQ.

**Figure 16:**
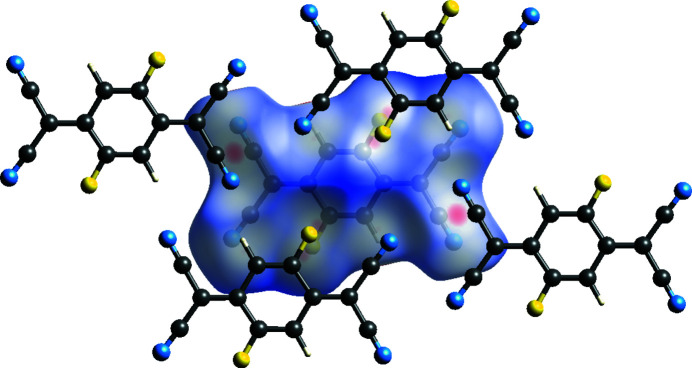
Hirshfeld surface of F_2_TCNQ, showing the close contacts between layers, with adjacent mol­ecules shown.

The layered arrangement of mol­ecules in F_2_TCNQ (Fig. 2 ▸) is similar to F_4_TCNQ polymorph II, with layers at a distance of 2.9275 (2) Å with respect to each other. The main difference between F_2_TCNQ and polymorph II is a change in the orientation of the mol­ecules in adjacent layers (Fig. 15 ▸). This change in orientation precludes the formation of the four-membered C≡N⋯C—F close contact ring motif observed in polymorph II. Instead, as seen in the Hirshfeld surface (Fig. 16 ▸), a C—F⋯C—F four-membered close contact motif is formed. A similar four-membered ring of close contacts is also seen between two cyano groups in adjacent layers in this structure. As F_2_TCNQ contains H atoms, hydrogen bonds can and do form, with C—H⋯N≡C contacts forming between the mol­ecules within layers.

Pairwise inter­action energies were calculated for CSD refcode BERZON03 (Krupskaya *et al.*, 2015 ▸; Table S4 in the supporting information). There are two different inter­acting modes between mol­ecules in adjacent layers to a central mol­ecule, unlike in polymorph II where there is only one. Similarly, there are two types of inter­action to the central mol­ecule from mol­ecules in the same layer – one set of mol­ecules that forms hydrogen bonds and another which has no close contacts; these are coloured in Fig. S13 (see supporting information).

Like polymorph II, the largest pairwise inter­action energy is calculated between mol­ecules in adjacent layers to the mol­ecule with a C—F four-membered ring motif and a C—H⋯N close contact. This value is smaller than the inter-layer inter­action of polymorph II (−29.0 *versus* 33.0 kJ mol^−1^). The calculated energy of dispersion in this pair is larger than in polymorph II; however, the electrostatic energy is much smaller. Mol­ecules that form close contacts to hydrogen, found within the layers, give the next largest value (−26.4 kJ mol^−1^). The smallest value corresponds to the other mol­ecule within the layer, which forms no close contacts to the central mol­ecule. The average energy for the surrounding inter­actions to the central mol­ecule is calculated as −21.0 kJ mol^−1^.

### Structure of F_4_TCNQ–toluene solvate   

The F_4_TCNQ and toluene mol­ecules lie in layers perpendicular to the [101] direction; further details are in the supporting information (§S3).

## Summary   

The results reported here provide a clear example of polymorphism in F_4_TCNQ. A second polymorph of F4TCNQ, polymorph II, was grown concomitantly alongside the previously known polymorph I from a saturated solution of DCM. Pairwise inter­action energies calculated in *CrystalExplorer* show that both structures have similar total energies – with polymorph II being the lowest, suggesting that polymorph II may be the more thermodynamic polymorph. Polymorph II exhibits a layered structure, with one mol­ecule in the reduced unit cell, which has been suggested to promote electron mobility and charge transfer (Chernyshov *et al.*, 2017 ▸). The structure is also very similar to the reported structure of F_2_TCNQ, which does possess such properties. Further study of this polymorph could provide new insights into charge mobility in this family of com­pounds.

## Supplementary Material

Crystal structure: contains datablock(s) polymorph_i, polymorph_ii, toluene_solvate, global. DOI: 10.1107/S2053229621006252/ef3019sup1.cif

Structure factors: contains datablock(s) polymorph_i. DOI: 10.1107/S2053229621006252/ef3019polymorph_isup2.hkl

Structure factors: contains datablock(s) polymorph_ii. DOI: 10.1107/S2053229621006252/ef3019polymorph_iisup3.hkl

Structure factors: contains datablock(s) toluene_solvate. DOI: 10.1107/S2053229621006252/ef3019toluene_solvatesup4.hkl

Supporting information file. DOI: 10.1107/S2053229621006252/ef3019polymorph_isup5.cml

Supporting information file. DOI: 10.1107/S2053229621006252/ef3019toluene_solvatesup6.cml

Additional figures, tables and structural information. DOI: 10.1107/S2053229621006252/ef3019sup7.pdf

CCDC references: 2090530, 2090529, 2090528

## Figures and Tables

**Table 1:** Experimental details

	F_4_TCNQ polymorph I	F_4_TCNQ polymorph II	F_4_TCNQ–toluene solvate
Crystal data			
Chemical formula	C_12_F_4_N_4_	C_12_F_4_N_4_	C_12_F_4_N_4_·C_7_H_8_
*M*_r_	276.16	276.16	368.29
Crystal system, space group	Orthorhombic, *P* *b* *c* *a*	Orthorhombic, *P* *n* *n* *m*	Monoclinic, *P*2_1_/*c*
Temperature (K)	100	100	150
*a*, *b*, *c* (Å)	9.1799 (3), 8.0482 (3), 14.5541 (5)	7.5140 (4), 11.6787 (6), 5.9347 (3)	8.1314 (2), 7.4141 (2), 13.6796 (4)
α, β, γ (°)	90, 90, 90	90, 90, 90	90, 100.551 (3), 90
*V* (Å^3^)	1075.28 (6)	520.79 (5)	810.76 (4)
*Z*	4	2	2
Radiation type	Ag *K*α, λ = 0.56086 Å	Ag *K*α, λ = 0.56086 Å	Cu *K*α
μ (mm^−1^)	0.09	0.10	1.09
Crystal size (mm)	0.28 × 0.22 × 0.16	0.3 × 0.17 × 0.12	0.41 × 0.05 × 0.03

Data collection			
Diffractometer	Bruker Photon II CPAD	Bruker Photon II CPAD	Rigaku OD Xcalibur Atlas Gemini ultra
Absorption correction	Numerical (*SADABS*; Bruker, 2016 ▸)	Numerical (*SADABS*; Bruker, 2016 ▸)	Analytical [*CrysAlis PRO* (Rigaku OD, 2015 ▸), based on expressions derived by Clark & Reid (1995 ▸)]
*T*_min_, *T*_max_	0.919, 0.982	0.931, 0.974	0.806, 0.975
No. of measured, independent and observed [*I* > 2σ(*I*)] reflections	189710, 5103, 4271	115983, 2628, 2285	10970, 1433, 1194
*R*_int_	0.043	0.043	0.045
(sin θ/λ)_max_ (Å^−1^)	1.043	1.043	0.597

Refinement			
*R*[*F* ^2^ > 2σ(*F* ^2^)], *wR*(*F* ^2^), *S*	0.034, 0.115, 1.09	0.031, 0.111, 1.07	0.039, 0.109, 1.08
No. of reflections	5103	2628	1433
No. of parameters	91	61	155
No. of restraints	0	0	161
H-atom treatment	–	–	H-atom parameters constrained
Δρ_max_, Δρ_min_ (e Å^−3^)	0.75, −0.33	0.77, −0.26	0.44, −0.21

Computer programs: *APEX2* (Bruker, 2009 ▸), *CrysAlis PRO* (Rigaku OD, 2015 ▸), *SAINT* (Bruker, 2009 ▸), *SHELXT* (Sheldrick, 2015*a* ▸), *SHELXL2014* (Sheldrick, 2015*b* ▸), *OLEX2* (Dolomanov *et al.*, 2009 ▸) and *XPREP* (Bruker, 2009 ▸).
