# Supplementary material for: Structural investigations into a new polymorph of F4TCNQ: towards enhanced semiconductor properties
Source: Acta Crystallogr C Struct Chem. 2021 Jun 28;77(Pt 7):426–34. doi: 10.1107/S2053229621006252 (PMC8254529; doi:10.1107/S2053229621006252)
Supplement: Supplementary file 7 [file c-77-00426-sup7.pdf]

STRUCTURAL CHEMISTRY

Volume 77 (2021)

Supporting information for article:

Structural investigations into a new polymorph of F4TCNQ: towards enhanced semiconductor properties

Natalie T. Johnson, Michael R. Probert and Paul G. Waddell

S1. Additional Information

Structure overlay of the two structures: The root mean square of the deviation when overlaying one asymmetric unit on top of the other is 0.023 Å, an exceptionally low value which indicates negligible conformational variation.

S1.1. Additional images

S1.1.1. Hirshfeld surfaces of F_4TCNQ Polymorph I

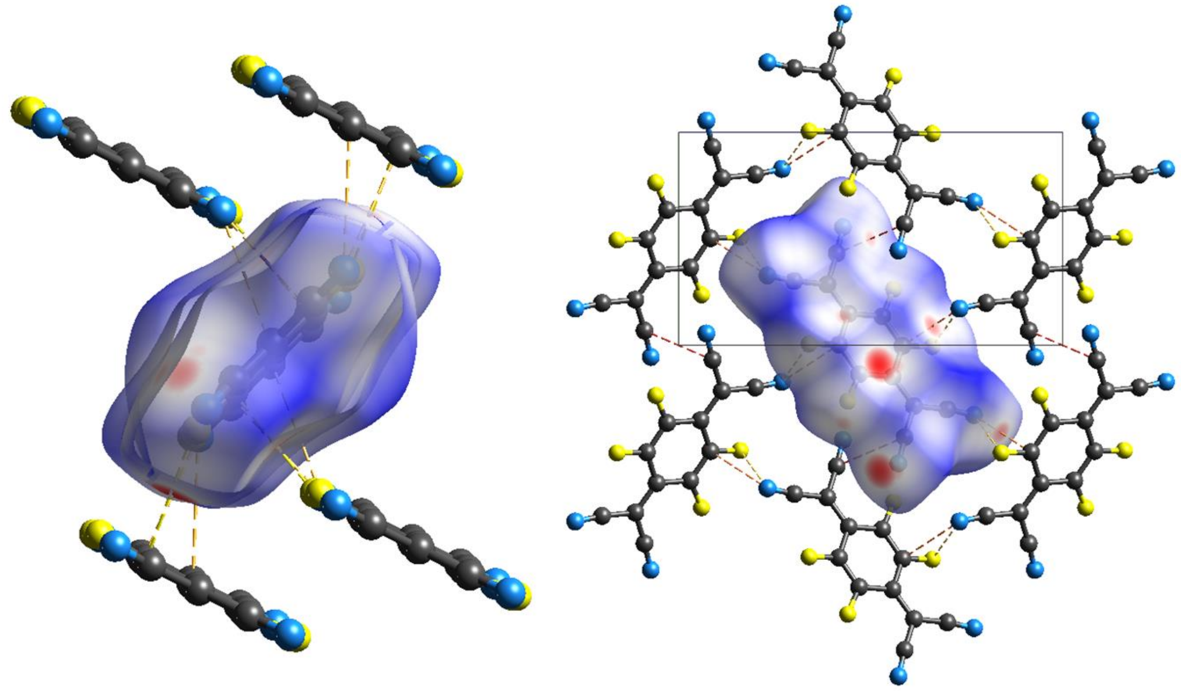

Figure S1 Hirshfeld surface and close contacts polymorph I, with adjacent molecules shown.

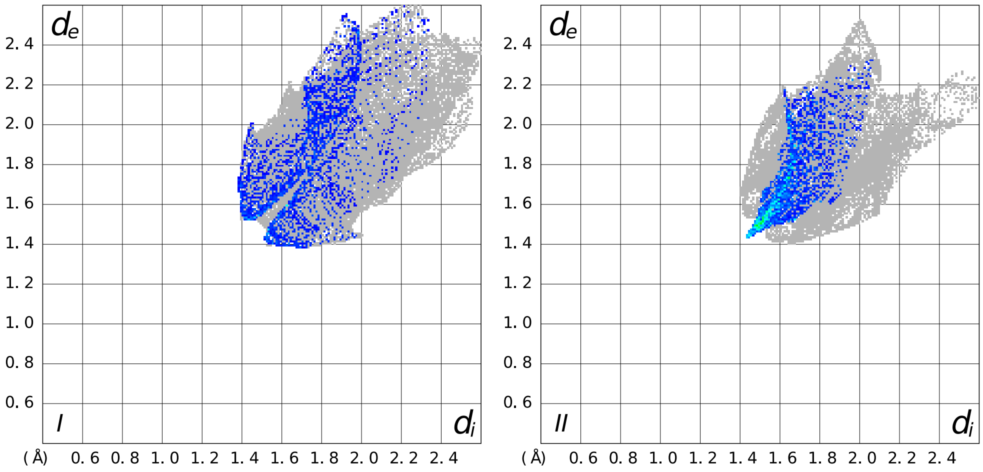

Figure S2 Fingerprint plots showing only $F_{internal}\cdots F_{external}$ contacts for polymorphs I and II.

S1.1.2. F_2TCNQ Hirshfeld surfaces

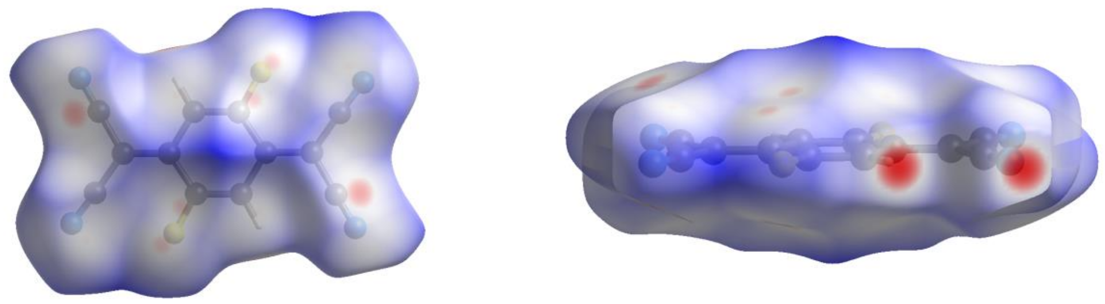

Figure S3 Hirshfeld surfaces for F_2TCNQ indicating large red areas on nitrogen and hydrogen where N···H hydrogen bond formation takes place.

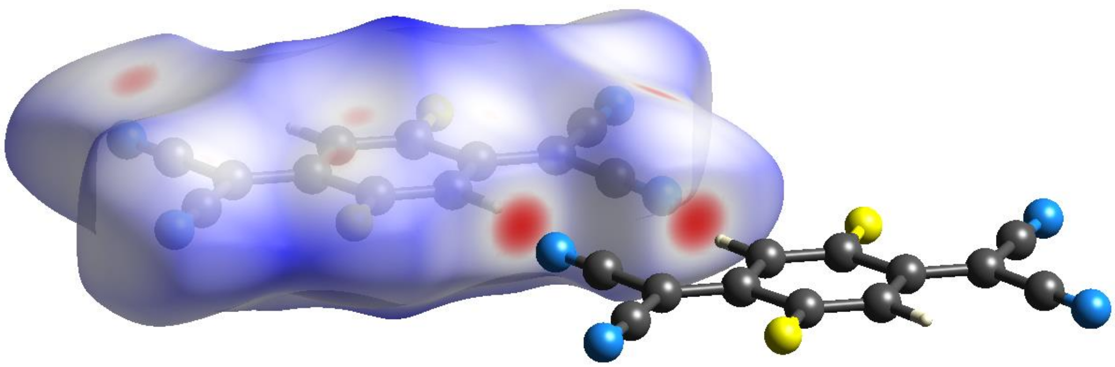

Figure S4 Hirshfeld surface showing hydrogen bonding within layer.

S1.2. F_2TCNQ Fingerprint plots

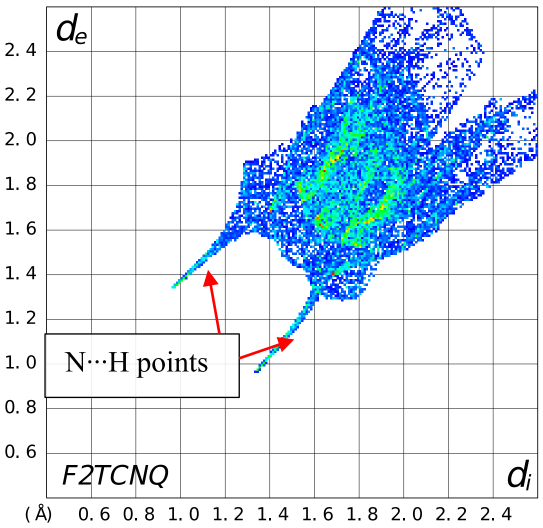

Figure S5 Fingerprint plot of F_2TCNQ. This plot shows the presence of hydrogen bonding, which is not formed in F_4TCNQ (as no hydrogen atoms are present).

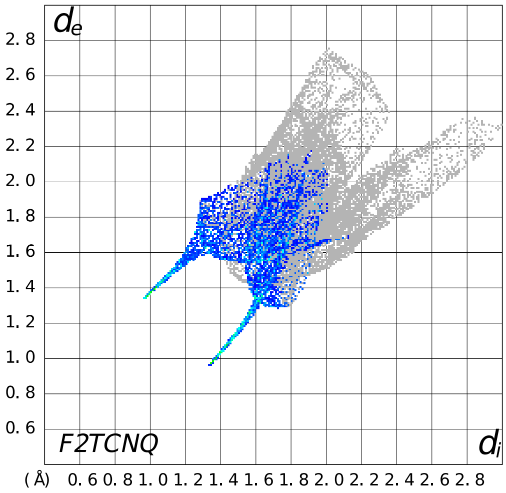

Figure S6 Fingerprint plot for F_2TCNQ showing only N···H contacts.

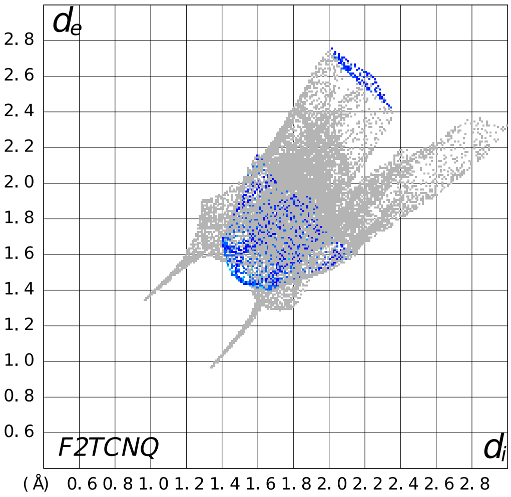

Figure S7 Fingerprint plot for F_2TCNQ showing only F···F contacts.

S1.2.1. Closest atom pair comparison of fingerprint plots for F_4TCNQ polymorph I, II and F_2TCNQ.

The percentages of the closest pairs of atoms by element from the fingerprint plot can be plotted for each polymorph of F_4TCNQ and F_2TCNQ for further structural comparisons (Figure S8). Both polymorphs of F_4TCNQ have mostly similar ratios of contacts between elements. It could be noted that there is a higher percentage of contacts between the same atoms in polymorph II (F···F, N···N and C···C), however the differences between percentages are small in all cases (range: 0.2-5.2 % (Table S1)). The values percentages for F_2TCNQ are mostly comparable to F_4TCNQ apart from the reduced % of F···N closest contacts. N···H closest contacts make up the second largest proportion. This may be expected there are hydrogen atoms in the F_2TCNQ molecule, allowing hydrogen bonds between hydrogen and nitrogen to be formed in this structure, a generally structure-directing feature, where they are absent in the structures of F_4TCNQ. The plot also indicates a small % of C···H closest contacts, although there appear to be no H···H or F···H closest contacts.

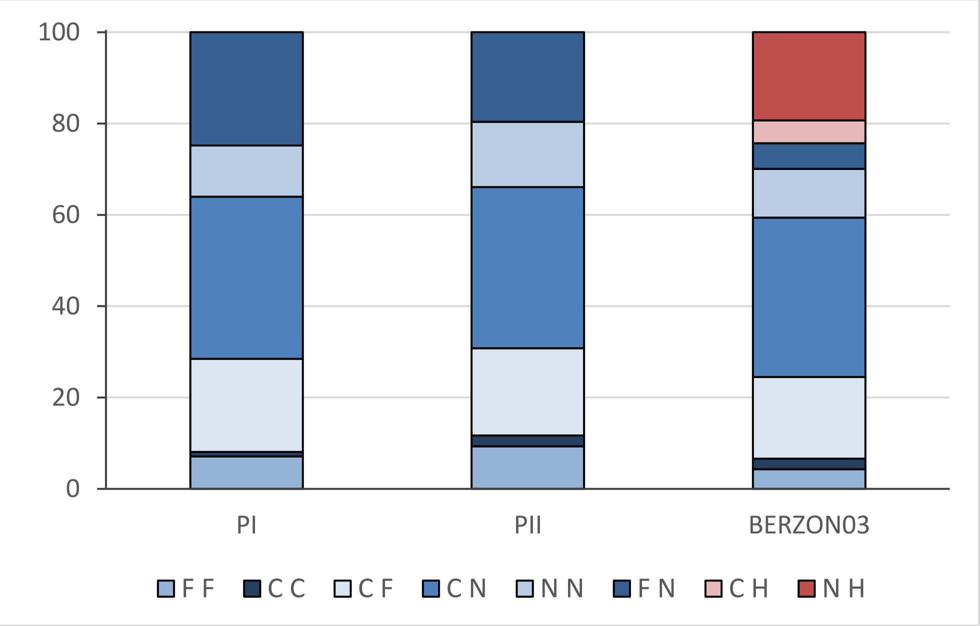

Figure S8 % of closest atom pairs by element calculated from fingerprint plots of molecules.

Reciprocal		Polymorph I	Polymorph II	Difference	BERZON03
F	F	7.1	9.3	2.2	4.3
C	C	1	2.4	1.4	2.3
C	F	20.4	19.1	-1.3	17.9
C	N	35.5	35.3	-0.2	34.9
N	N	11.2	14.3	3.1	10.7
F	N	24.8	19.6	-5.2	5.6
F	H	-	-	-	0
C	H	-	-	-	5
N	H	-	-	-	19.3
H	H	-	-	-	0

Table S1 Percentages of in the calculation of Figure S8: '% of closest atom pairs by element from fingerprint plots of molecules'.

S1.3. Energy calculations

S1.3.1. F_4TCNQ polymorph I

Table S2 shows the contributions to the total energy for each independent pairwise interaction energy for polymorph I. The coloured number in column 1 corresponds to the colour of the atoms shown in Figure S9 which illustrates the different molecular environments surrounding the central molecule.

N	Symmetry operation	R	E_{ele}	E_{pol}	E_{dis}	E_{rep}	E_{tot}
4	x+1/2, -y+1/2, -z	6.10	-8.3	-4.8	-28.8	24.1	-22.5
2	x, y, z	8.04	-20.5	-3.8	-23.7	17.9	-34.0

4	-x, y+1/2, -z+1/2	8.31	-7.1	-2.0	-15.4	12.1	-15.0
4	-x+1/2, -y, z+1/2	8.60	-3.3	-1.0	-5.6	1.8	-8.0

Table S2 Calculated energies between molecules in F_4TCNQ for polymorph I (kJ/mol). Colour of number, N, corresponds to the colour of a molecule within the preceding Figure S9.

There are 4 different molecular environments surrounding the central molecule, two within the layers and two in adjacent layers. Molecules within adjacent layers that are not in the herringbone pattern relative to the central molecule (purple) have the lowest E_{tot}. These molecules also do not have any close contacts to the central molecule. The largest E_{tot} is calculated for molecules within the same layer that are arranged parallel to each other. These molecules exhibit the close C···C contact resulting from overlap of the C(5) atoms with each other. These molecules have a large electrostatic energy between them – although, as the close contact is between the same atom in both molecules, it is unlikely to be electrostatically favourable. Other factors must contribute to the low electrostatic energy. Figures S10 and S11 show energy frameworks for $E_{tot.}$

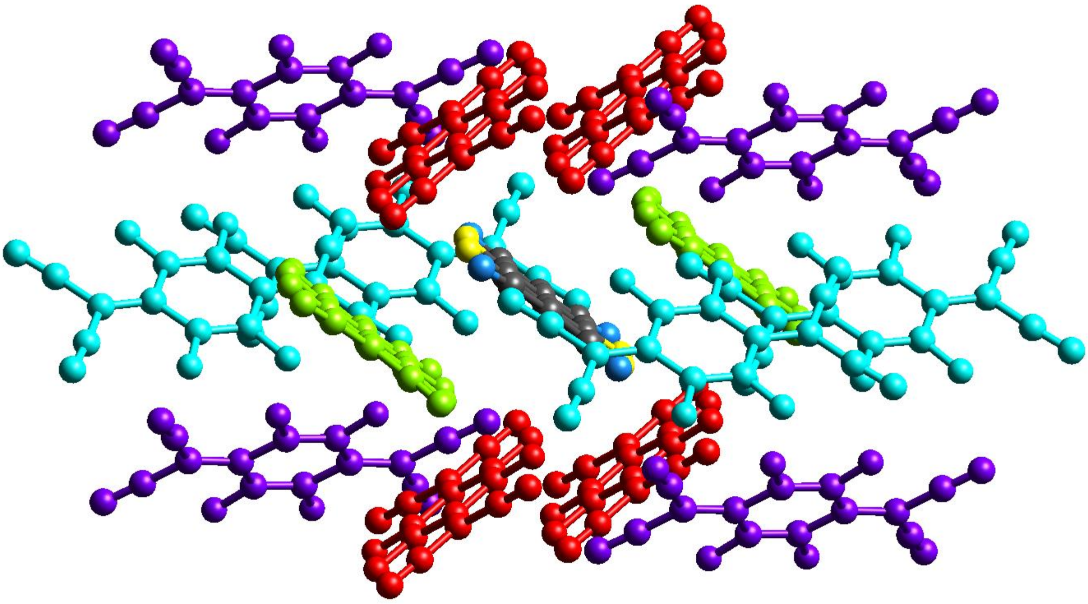

Figure S9 Colour coordinated contributions to interaction energy from polymorph I. Colour of molecule corresponds to the colour of N in Table S2.

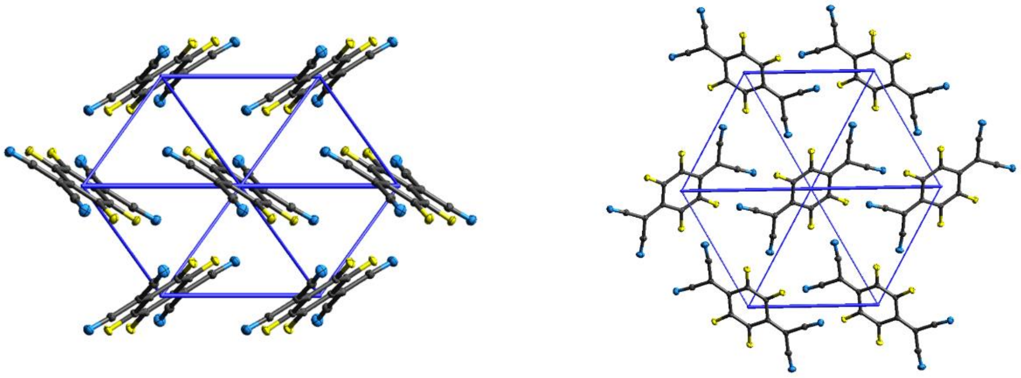

Figure S10 Energy frameworks for polymorph I within ‘layers’. 1. Just molecules in herringbone’, 2. Molecules within layer

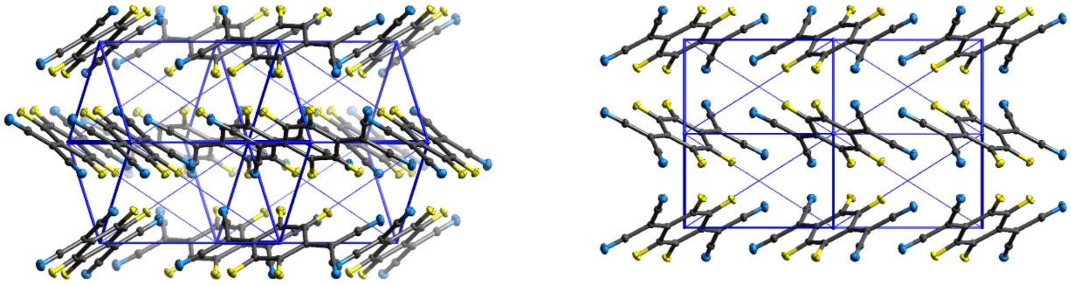

Figure S11 More alternative views of energy frameworks for polymorph I. 1. A view of Figure 14 with no pairwise energies removed, 2. An alternative direction.

S1.3.2. F_4TCNQ polymorph II

Table S3 shows the pairwise interaction energies for polymorph II, with the corresponding molecular diagram in Figure S12. There are 3 different crystal packing environments around the central molecule, 2 sets within a layer (red and blue) as well as one set of 8 atoms in adjacent layers (green). Molecules in adjacent layers, where in the Hirshfeld surface there were a number of close contacts (Figure 7) – including the possibility of a four membered ring of electrostatically attractive atom close contacts, are calculated as having large total energies. Molecules within layers, however, only provide a very small contribution to total energy. Interestingly, the molecules within the layers are the only ones that have a repulsive electrostatic contribution to the total energy in either polymorph. The lowest contribution is from the molecules rendered in red, where in Hirshfeld surfaces there is close contact between the fluorine atoms of adjacent molecules, although the total energy is still slightly attractive (-1.1 kJmol^{-1}) due to the contribution from the dispersion energies.

N	Symmetry operation	R	E_{ele}	E_{pol}	E_{dis}	E_{rep}	E_{tot}
2	x, y, z	7.51	8.6	-0.9	-14.1	4.5	-1.1
8	x+1/2, -y+1/2, -z+1/2	7.55	-18.5	-4.1	-26.5	19.9	-33.3
2	x, y, z	11.68	0.5	-0.6	-6.7	3.8	-3.5

Table S3 Interaction energies calculated for polymorph II (kJ/mol^{-1}). Colour of number corresponds to the colour of a molecule within the preceding Figure S12.

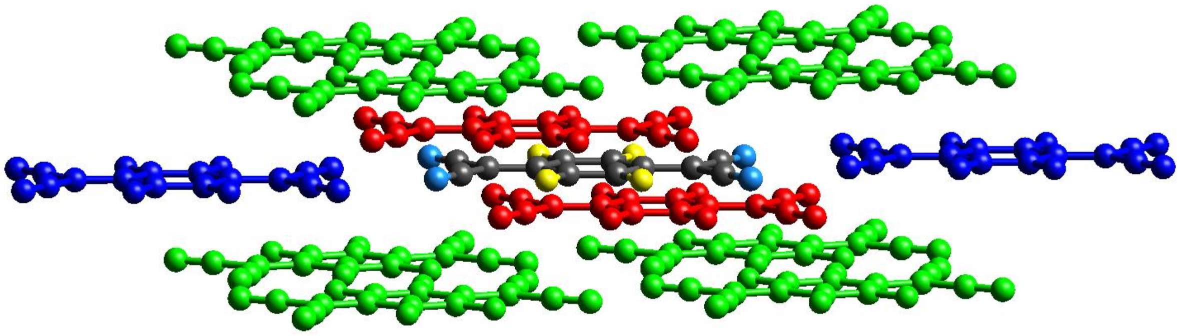

Figure S12 Colour coordinated contributions to interaction energy for polymorph II. The colour of the molecule corresponds to the colour of N in Table S3.

These total energies for the pairs were then used to calculate the average energy for the crystal structure, as the sum of total energy for N_j equivalent molecules:

$$E_{avg} = \frac{\sum_j N_j \times E_{tot,j}}{\sum_j N_j} \qquad \text{Equation S1}$$

S2. F$_2$TCNQ

Pairwise energies are calculated with CCD-BERZON03 as this structure was measured at the same temperature as the collections in the paper. The green molecules, illustrated in Figure S13, have the largest total energy − 29.0 kJmol^{-1} (Table S4). This is comparable to − 33.3 kJmol^{-1} as calculated for molecules in adjacent layers of F$_4$TCNQ polymorph II. However, there is a much greater contribution to the energy of F$_2$TCNQ from molecules within the layers, owing to the hydrogen bonding present within the structure.

N	Symmetry operation	R	E_{ele}	E_{pol}	E_{dis}	E_{rep}	E_{tot}
2	x, y, z	11.37	-1.8	-1.0	-8.9	7.8	-5.6
4	x+1/2, y+1/2, z	5.87	-9.2	-4.9	-33.2	21.6	-29.0

2	x, y, z	8.84	-28.4	-4.5	-12.1	28.2	-26.4
4	x+1/2, y+1/2, z	9.29	-11.8	-1.9	-16.3	16.3	-18.0

Table S4 Pairwise interaction energies for F_2TCNQ (kJ/mol). Colour of N in table corresponds to colour of a molecule in Figure S13.

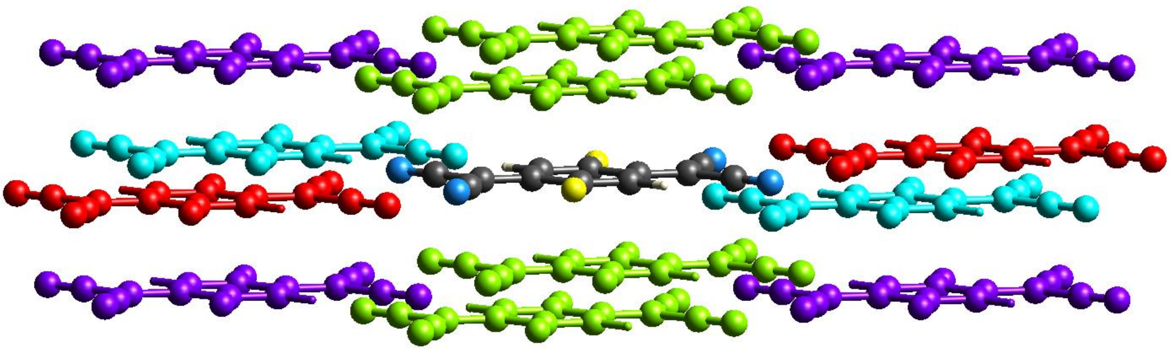

Figure S13 Colour coded contributions to pairwise interaction energy for F_2TCNQ. The colour of the molecule corresponds to the colour of N in Table S4.

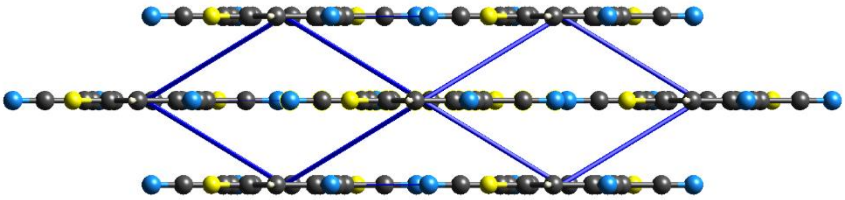

Figure S14 Energy framework calculated for F_2TCNQ viewed along the [100] direction.

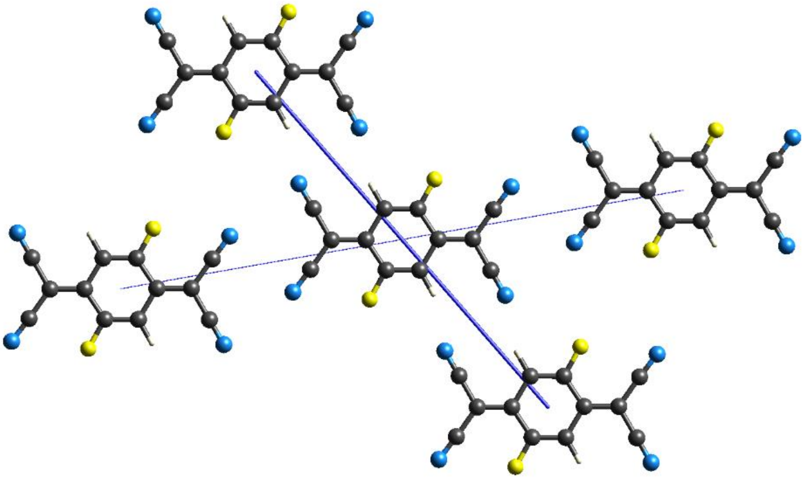

Figure S15 View along the [010] direction in the structure of F_2TCNQ, showing the total energy framework for molecules within the layer. There is a larger E_{tot} for those molecules between which hydrogen bonds are observed.

S3. F_4TCNQ·Toluene Solvate structure

Slow evaporation of solvent from a saturated solution of F_4TCNQ in toluene produced red needle-shaped single crystals that were determined to be of a toluene solvate of F_4TCNQ.

A crystal of F_4TCNQ·toluene was measured using Cu radiation ($\lambda_{CuK\alpha}$ = 1.54184 Å) at 150 K on a Rigaku Oxford Diffraction Xcalibur Atlas Gemini diffractometer equipped with an OxfordCryosystems N_2 open-flow cooling device.

Diffraction frames for F_4TCNQ·toluene solvate crystal were integrated and scaled using *CrysAlisPro* (Rigaku Oxford Diffraction, 2006). Intensities were corrected for absorption using a multi-faceted crystal model created by indexing the faces of the crystal for which data were collected. The structure solution and refinement were performed using *XL* and *XT* respectively within the Olex2 GUI.

Structure solution and refinement of the collected data yielded a structure containing both F_4TCNQ and toluene molecules. The crystal packs in the centrosymmetric $P2_1/c$ space group. Experimental details are included in Table S5.

The structure is comprised of discrete homomolecular layers, parallel to the crystallographic [100] plane, with the rings of the toluene and F_4TCNQ molecules stacked in an alternating sequence (Figure S16). Within the crystal structure, the toluene molecules are modelled as being disordered over two positions. The arrangement of the two molecules suggests π- π interactions between the rings of the two molecules.

Table S5 F_4TCNQ-Toluene solvate experimental details

Crystal data	
Chemical formula	$C_{12}F_4N_4 \cdot C_7H_8$
M_r	368.29
Crystal system, space group	Monoclinic, $P2_1/c$
Temperature (K)	150
a, *b*, *c* (Å)	8.1314 (2), 7.4141 (2), 13.6796 (4)
β (°)	100.551 (3)
V (Å^3)	810.76 (4)
Z	2
Radiation type	Cu *K*α
μ (mm^{-1})	1.09
Crystal size (mm)	0.41 × 0.05 × 0.03
Data collection	
T_{min}, T_{max}	0.806, 0.975
No. of measured, independent and observed [$I > 2\sigma(I)$] reflections	10970, 1433, 1194
R_{int}	0.045
$(\sin \theta/\lambda)_{max}$ (Å^{-1})	0.597
Refinement	

$R[F^2 > 2\sigma(F^2)]$, $wR(F^2)$, S	0.039, 0.109, 1.08
No. of reflections	1433
No. of parameters	155
No. of restraints	161
H-atom treatment	H-atom parameters constrained
$\Delta\rho_{max}$, $\Delta\rho_{min}$ (e Å^{-3})	0.44, −0.21

Computer programs: *CrysAlis PRO* 1.171.38.42b (Rigaku OD, 2015), ShelXT (Sheldrick, 2015), XL (Sheldrick, 2008), Olex2 (Dolomanov *et al.*, 2009).

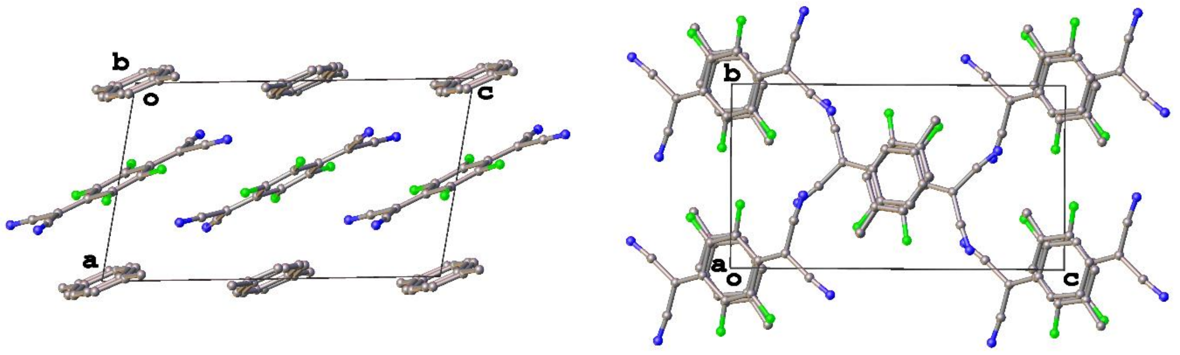

Figure S16 Packing in the crystal structure of the F_4TCNQ-toluene solvate.
